# Transcriptional suppression of Dicer by HOXB‐AS3/EZH2 complex dictates sorafenib resistance and cancer stemness

**DOI:** 10.1111/cas.15319

**Published:** 2022-03-15

**Authors:** Chi‐Feng Tseng, Li‐Tzong Chen, Horng‐Dar Wang, Yi‐Hong Liu, Shine‐Gwo Shiah

**Affiliations:** ^1^ Graduate Program of Biotechnology in Medicine NTHU & NHRI Zhunan Taiwan; ^2^ 34881 Institute of Biotechnology National Tsing Hua University Hsinchu Taiwan; ^3^ 50115 National Institute of Cancer Research National Health Research Institutes Zhunan Miaoli County Taiwan; ^4^ Department of Oncology National Cheng Kung University Hospital College of Medicine National Cheng Kung University Tainan Taiwan; ^5^ Department of Internal Medicine Kaohsiung Medical University Hospital Kaohsiung Medical University Kaohsiung Taiwan; ^6^ Program in Environmental and Occupational Medicine Kaohsiung Medical University Kaohsiung Taiwan

**Keywords:** Dicer, EZH2, HOXB‐AS3, liver cancer, sorafenib resistance

## Abstract

Sorafenib is a multikinase inhibitor for the standard treatment of advanced liver cancer patients. However, acquired resistance to sorafenib is responsible for a poor prognosis. Therefore, uncovering the molecular mechanisms underlying sorafenib sensitization can provide biomarkers for sorafenib treatment and improve sorafenib activity in a precise medication. Here, we report that epigenetic suppression of Dicer by the *HOXB*‐*AS3*/EZH2 complex is responsible for sorafenib resistance. We observed that Dicer expression is inversely correlated with EZH2 levels, *HOXB*‐*AS3* expression, sorafenib resistance, and cancer stem cell properties in liver cancer patients. Furthermore, ectopic expression of Dicer induced liver cancer cells resensitization to sorafenib. Mechanistically, we found *HOXB*‐*AS3* physically interacts with EZH2 and recruits EZH2 to the *Dicer* promoter, resulting in epigenetic suppression of Dicer expression. These findings reveal that *HOXB*‐*AS3*/EZH2 complex–mediated Dicer suppression plays an important role in sorafenib resistance and cancer stemness and provide potential therapeutic strategies for diagnosing and treating liver cancer patients.

## Graphical abstract

*HOXB‐AS3/EZH2* complex–mediated Dicer suppression plays an important role in sorafenib resistance and cancer stemness and provides potential therapeutic strategies for diagnosing and treating liver cancer patients.

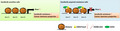

**Abbreviations**
ChIP: chromatin immunoprecipitation
EZH2: enhancer of zeste homolog 2
H3K27me3: trimethylation on Lys 27 of histone H3
HOXB‐AS3: HOXB cluster antisense RNA 3
lncRNAs: long noncoding RNAs
miRNA: microRNA
OCT4: octamer‐binding transcription factor 4
PcG: polycomb group
qRT‐PCR: quantitative reverse‐transcription polymerase chain reaction
CSC: cancer stem cell
shRNA: small‐hairpin RNA
SOX2: sex‐determining region Y‐box 2

## 1 INTRODUCTION

Sorafenib, an oral multikinase inhibitor that blocks the Ras/Raf/MEK/ERK signaling axis by inhibiting Raf kinase activity,^1^, ^2^ is the first clinically approved target therapy for advanced hepatocellular carcinoma (HCC) patients.^2^, ^3^ However, the low response rate and acquired sorafenib resistance are critical issues.^4^, ^5^ Thus, investigations into the molecular mechanisms of sorafenib resistance are urgently needed to develop novel therapeutic strategies for treating liver cancer patients.

Dicer is a cytoplasmic RNase III endonuclease that is crucial for miRNA maturation.^6^ Downregulation of Dicer is involved in tumorigenesis via a global decrease in miRNA expression^7^ and is associated with a poor prognosis and chemoresistance in many types of cancer.^8^, ^9^, ^10^, ^11^ Substantial evidence suggests that there are several potential regulators of Dicer, including the transcription factors MITF^12^ and Tap63^13^ as well as miR‐103/107.^14^ However, few studies have investigated the epigenetic regulation of Dicer.

Polycomb group (PcG) proteins are epigenetic gene silencers that are associated with tumorigenesis in numerous cancers.^15^ polycomb repressive complex 2 (PRC2) is present in the core of PcG in humans, consisting of three subunits including embryonic ectoderm development (EED), suppressor of zeste 12 homolog (SUZ12), and enhancer of zeste homolog 2 (EZH2) and catalyzes trimethylation on Lys 27 of histone H3 (H3K27me3).^16^ EZH2 overexpression is a poor prognostic marker in various cancers, including liver cancer, and targeting EZH2 is an anticancer strategy.^15^, ^17^, ^18^ In fruit flies, EZH2 interacts with sequence‐specific binding proteins that recognize polycomb response elements (PREs).^19^ In mammals, there are no consensus motifs, although EZH2 can bind to CpG‐rich domains, and some PcG recruiters can interact with EZH2 at selected target loci.^20^ Long noncoding RNAs (lncRNAs) can affect the epigenetic status and expression levels of many target genes by interacting with histone modifiers, chromatin‐remodeling complexes, transcriptional regulators, or the DNA methylation machinery.^21^ Recently, lncRNAs have been suggested to act as PcG recruiters and catalyze H3K27me3.^22^

In this study, we describe a novel mechanism by which lncRNA *HOXB*‐*AS3* acts as a PcG recruiter and promotes binding between EZH2 and the *Dicer* promoter. We also found that Dicer expression was inversely correlated with *HOXB*‐*AS3* and EZH2 expression, and negatively correlated with cancer stem cell (CSC) properties in liver cancer patients. Our findings suggest that suppression of Dicer by the *HOXB*‐*AS3*/EZH2 complex plays a critical role in sorafenib resistance and cancer stemness in liver cancer.

## 2 MATERIALS AND METHODS

### 2.1 Cell lines

Hep3B, HepG2, skHep1, C3A, and 293T were purchased from the American Type Culture Collection (ATCC). Huh7 and Huh1 were purchased from the Japanese Collection of Research Bioresources Cell Bank (JCRB). HA22T/VGH and PLC/PRF/5 were obtained from the Bioresource Collection and Research Center (BCRC) of the Food Industry Research and Development Institute (Hsinchu, Taiwan). HCC36 and Mahlavu were kind gifts from Dr. Jang‐Yang Chang (National Cheng Kung University [NCKU], Tainan, Taiwan). These cells were confirmed by STR profiling at the BCRC and Center for Genomic Medicine, NCKU. The sorafenib‐resistant cell line was generated by continuously exposing parental cells to sorafenib (Sigma‐Aldrich) with a final maximum concentration of 10 μM.

### 2.2 Western blotting

Cells were lysed in RIPA lysis buffer containing protease inhibitor cocktail and 1 mM Na_3_VO_4_. The protein samples were loaded onto SDS‐polyacrylamide gel for electrophoresis and then transferred to a PVDF membrane (Millipore). The blots were incubated with specific primary antibodies and then with secondary antibodies; subsequently, the protein was visualized using the enhanced chemiluminescent detection method. The following primary antibodies were used: Dicer (ab14601/Abcam), Drosha (ab12286/Abcam), Ago2 (ab‐57113/Abcam), Exportin‐5 (ab57491/Abcam), TRBP (ab42018/Abcam), EZH2 (ab‐3748/Abcam), and α‐Tubulin (T‐5168; Sigma).

### 2.3 RNA isolation, RT‐PCR, and qRT‐PCR

Total RNAs were isolated from cells by TRIzol reagent (Roche). For reverse transcription, 10 μg total RNA, random hexamer primers (Roche Applied Science), and M‐MLV reverse transcriptase (Invitrogen) were used. Quantitative RT‐PCR was performed by the Lightcycler 480 system (Roche). The qRT‐PCR data were normalized to the level of *GAPDH*. The sequences of the PCR primer are shown in Table S1.

### 2.4 Flow cytometry analysis

Cells (5 × 10^5^) were harvested and resuspended in 50 μl of PBS and then incubated with anti‐CD44 antibody (PE, 130–098–210, Miltenyi Biotech) or anti‐CD133 antibody (PE, 130–098–826, Miltenyi Biotech) for 30 minutes at 4°C. After incubation, cells were analyzed by flow cytometry (FACSCalibur, BD Biosciences). Nonspecific mouse IgG antibody was used as isotype control for comparison.

### 2.5 Sphere formation assay

Cells were dissociated with trypsin‐EDTA and resuspended in serum‐free medium containing B27 supplement (Invitrogen), 20 ng/μl epidermal growth factor (EGF) (Invitrogen), and 10 ng/μl basic fibroblast growth factor (bFGF) (Invitrogen) to re‐form spheres. Cells were then seeded into an ultralow‐attachment 24‐well plate (Corning Inc) at a density of 1000 cells per well for 7‐21 days.

### 2.6 Luciferase reporter assay

Cells (50% confluent in 12‐well plates) were cotransfected with 1 μg of indicated *Dicer* promoter reporter gene constructs plus 0.1 μg of pRL‐TK plasmids using FuGENE (Roche) according to the manufacturer's instructions. Luciferase activities were determined by a dual‐luciferase reporter assay system (Promega) following the protocols provided by the manufacturer and analyzed by normalization of firefly luciferase activity to Renilla luciferase activity.

### 2.7 Chromatin immunoprecipitation (ChIP) assay

Cell lysates were sonicated to break DNAs to sizes of 300‐1000 bp. Protein and DNA complexes were precipitated by either nonimmune IgG or anti‐EZH2 or anti‐H3K27me3 (Millipore) overnight at 4°C with rotation. DNAs were then isolated from protein‐DNA complexes. The enrichment of DNA was analyzed by qRT‐PCR with the LightCycler FastStart DNA Master SYBR Green I kit (Roche). The sequences of the PCR primer are shown in Table S1.

### 2.8 RNA immunoprecipitation (RIP) and RNA pulldown assay

For RIP, the Magna RIP RNA‐binding protein immunoprecipitation kit (17–700, Millipore) was used according to the manufacturer's instructions. For RNA pulldown assay, in vitro synthesis of biotinylated RNA by T7 RNA polymerase was used to synthesize biotin‐labelled lncRNA. RNA‐bound beads were then equilibrated in protein lysate. Proteins were eluted using SDS‐PAGE loading buffer.

### 2.9 Animal studies

All animal work was done in accordance with a protocol approved by the Institutional Animal Care and Use Committee of the National Health Research Institutes. Four‐ to six‐week‐old CB‐17 severe combined immunodeficient (SCID) male mice (supplied by LASCO) were used for tumor growth in a xenograft study. A total of 5 × 10^6^ cells were subcutaneously (s.c.) injected into the dorsum of mice. When tumor volumes reached approximately 100 mm^3^, as determined by measuring tumor length and width using calipers and calculating volume through the formula (1/2 [length × width^2^]), mice were randomly divided into two groups to receive sorafenib (15 mg/kg/day) or vehicle by oral gavage, and tumor volumes were measured every 3 days.

### 2.10 Specimens

Tissues were obtained from the National Cheng Kung University Hospital with institutional review board approval (A‐ER‐103–174), and written informed consent was obtained from all patients. Total RNA was extracted from liver cancer tissues using High Pure FFPE RNA Micro Kit (Roche Applied Science).

### 2.11 MTT assay

Cell viability was assessed using the 3‐(4, 5‐dimethylthiazol‐2‐yl)‐2, 5‐diphenylterazolium bromide (MTT, Sigma‐Aldrich) method. Briefly, cells were seeded into 96‐well plates and then treated with the indicated sorafenib concentrations. MTT reagent (0.5 mg/ml) was added and then incubated for 4 hours. The dye absorbance was measured at a wavelength of 570 nm with background subtraction at 630 nm using an ELISA reader.

### 2.12 Statistical analysis

All data were analyzed using GraphPad Prism 6 software. Data were shown as the mean ± SD of three independent experiments. Statistical analysis among the experimental groups was conducted using the two‐tailed Student's *t* test for comparison between two groups. Differences with a *P*‐value of less than 0.05 were considered statistically significant.

## 3 RESULTS

### 3.1 Dicer enhances sorafenib sensitivity and decreases the cancer stemness of liver cancer cells in vitro and in vivo

**FIGURE 1:**
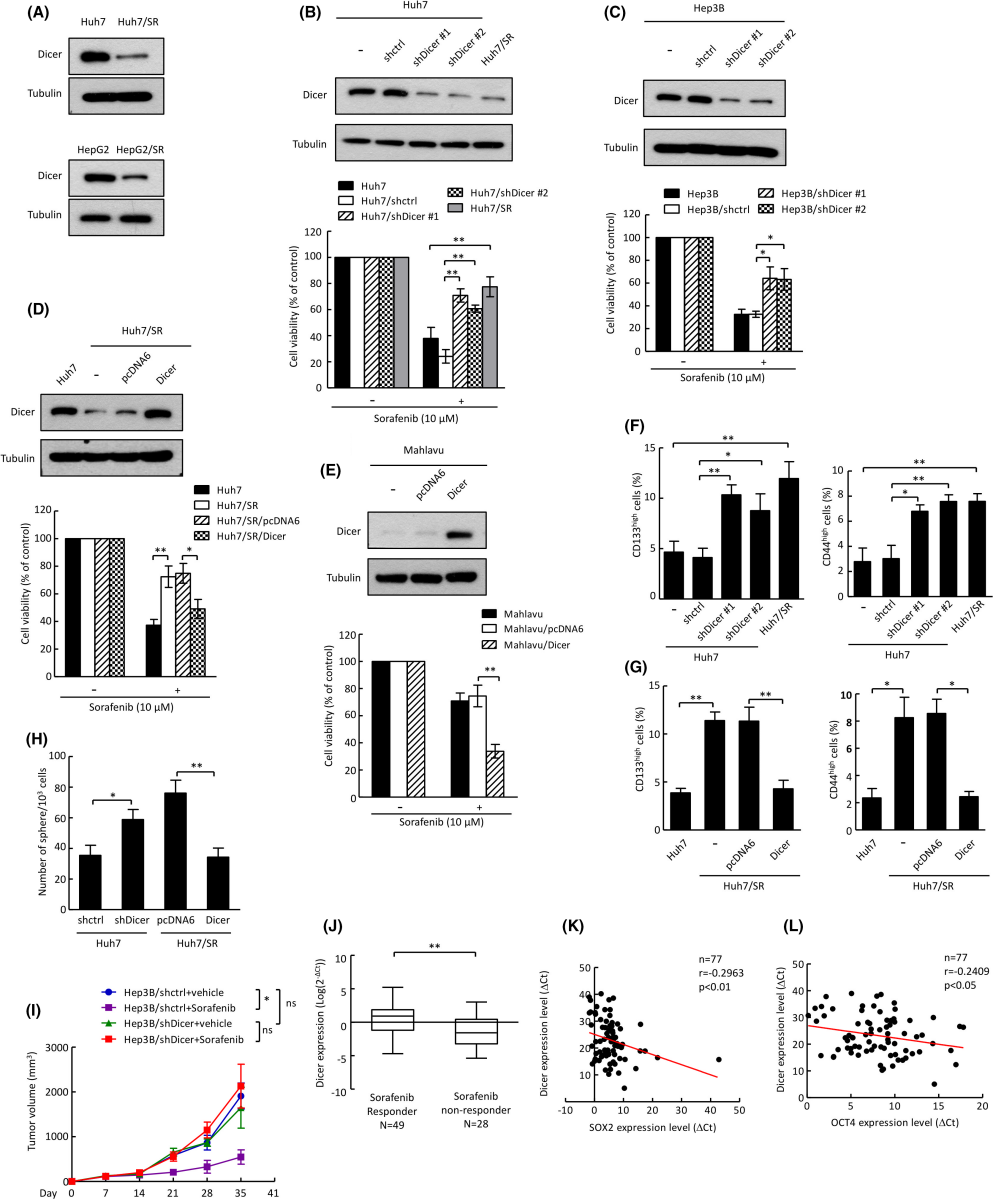
Dicer is crucial for sorafenib sensitization in liver cancer in vitro and in vivo. A‐E, Dicer expression was analyzed by Western blotting in indicated cells. Cell viability was analyzed using the MTT assay. F, G, CD133high (left) and CD44high (right) populations were analyzed using flow cytometry. H, Sphere formation ability was examined using sphere formation assay. Error bars indicate the mean ± SD of three independent experiments. *p < 0.05, **p < 0.01; Student's *t* test. I, Volume of the xenograft tumors formed by the indicated cells with sorafenib treatment. Error bars indicate the mean ± SD of six primary tumors. *p < 0.05; ns, not significant (*p* > 0.05); Student's *t* test. J, Dicer expression in liver cancer patients was analyzed by qRT‐PCR. **p < 0.01; Student's *t* test. K, L, Correlation between Dicer and SOX2 (K) and between Dicer and OCT4 (L) in liver cancer patients. Correlation coefficient (*r*), sample number (*n*), and *P*‐values are shown within the box plot

miRNA dysregulation is critical for physiopathological disorders, including drug resistance in cancer.^23^ Surprisingly, we found that most miRNAs are downregulated in the sorafenib‐resistant liver cancer cell line, Huh7/SR cells (generated from Huh7 cells by long‐term exposure to sorafenib), compared with Huh7 cells, according to miRNA microarray analysis (Figure S1A). We further detected the expression of miRNA processing enzymes and found that the expression of Dicer was decreased in both sorafenib‐resistant liver cancer cells (Huh7/SR and HepG2/SR cells) compared with their parental cells (Huh7 and HepG2 cells; Figure 1A and Figure S1B). In addition, Dicer expression was inversely correlated with the IC_50_ of sorafenib in liver cancer cells (Figure S1C). Transfection with small‐hairpin RNA (shRNA) specific against Dicer significantly increased the cell viability after sorafenib treatment (Figure 1B,C). Consistent with the shRNA results, overexpression of Dicer not only decreased the cell viability and the expression of p‐MEK and p‐ERK1/2 but also increased the cleavage of PARP‐1 in cells that were not sensitive to sorafenib after sorafenib treatment (Figure 1D,E and Figure S1D). We further analyzed the expression of CSC surface markers CD133 and CD44 in liver cancer cells by modulating Dicer expression. We found that knockdown of Dicer increased the CD133^high^ and CD44^high^ phenotypes in Huh7 and Hep3B cells (Figure 1F and Figure S1E). Consistently, overexpression of Dicer decreased the CD133^high^ and CD44^high^ phenotypes in Huh7/SR and Mahlavu cells (Figure 1G and Figure S1F). We also found that Dicer negatively regulated OCT4 and SOX2 expression in liver cancer cells based on qRT‐PCR analysis (Figure S1G‐J). Furthermore, sphere formation assay results demonstrated that Dicer decreased cancer stemness (Figure 1H and Figure S1K). To further study the role of Dicer in an in vivo model, we s.c. injected stable transfected cell lines (Hep3B/shctrl and Hep3B/shDicer) into the dorsum of SCID mice. The tumor volumes of Hep3B/shctrl‐bearing mice with sorafenib treatment were lower than in Hep3B/shDicer‐bearing mice, suggesting that depletion of Dicer reduces the efficiency of sorafenib (Figure 1I). We also used qRT‐PCR to detect Dicer expression in 77 liver cancer patients who received sorafenib treatment to evaluate the clinical significance of Dicer in liver cancer patients. All 77 patients received sorafenib treatment for at least 2 months and were divided into stable‐disease (SD), partial‐response (PR) and progressive‐disease (PD) groups based on the RECIST criteria 1.1.^24^ The SD and PR groups were sorafenib responders (*N* = 49), and the PD group was a sorafenib nonresponder group (*N* = 28). We found that sorafenib responders showed higher Dicer expression than sorafenib nonresponders (Figure 1J). We also found that Dicer expression was inversely correlated with SOX2 and OCT4 expression in liver cancer tissues (Figure 1K,L). Bioinformatics analyses using the Oncomine database showed that Dicer expression was decreased in tumor tissues compared with normal tissues and inversely correlated with the tumor grade and the presence of stem cell surface markers (CD133 and CD44; Figure S1L‐O). These findings demonstrate that Dicer suppresses the tumorigenesis of liver cancer and is involved in sorafenib sensitivity and cancer stemness.

### 3.2 EZH2‐mediated transcriptional suppression of Dicer contributes to sorafenib resistance in liver cancer

**FIGURE 2:**
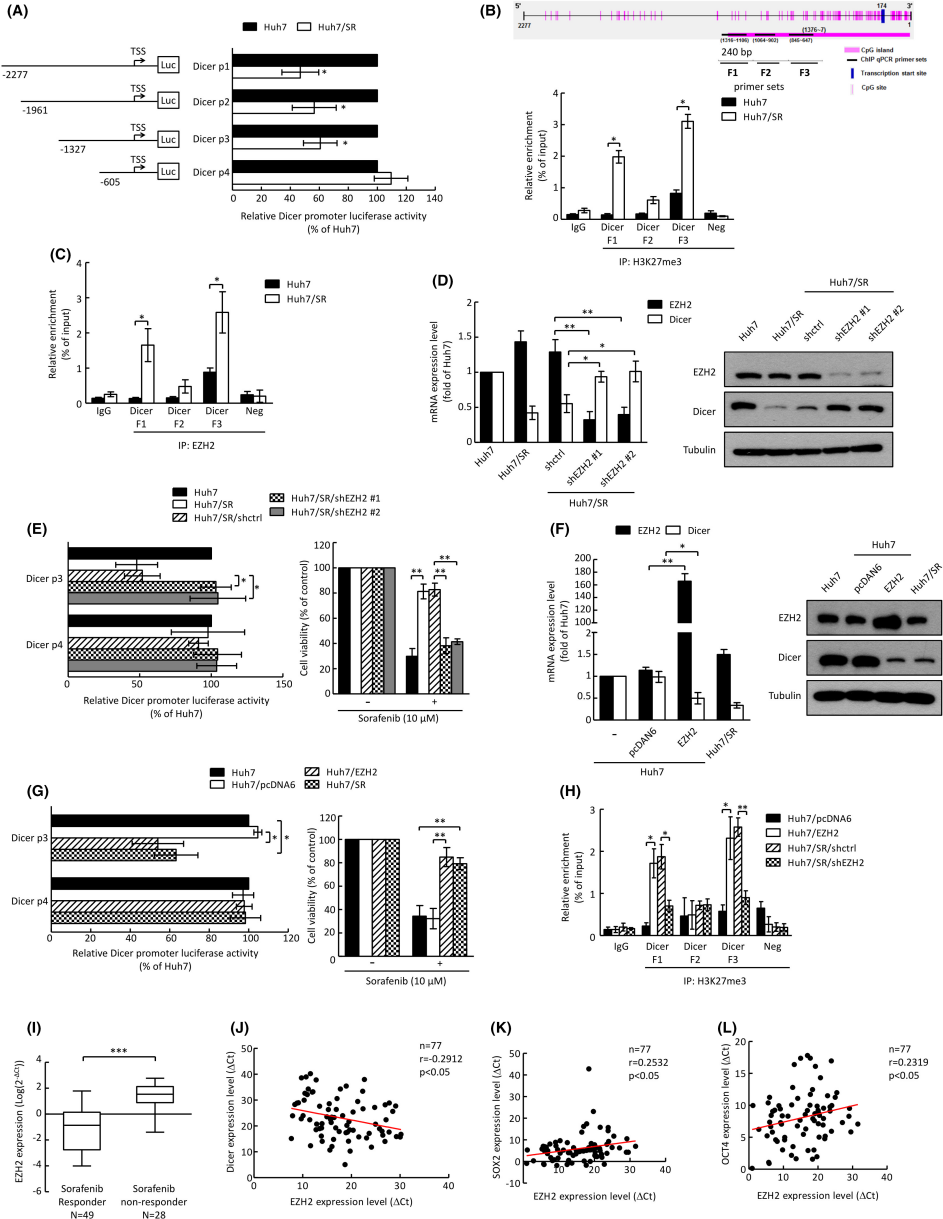
EZH2 transcriptionally suppresses Dicer in sorafenib‐resistant cells. A, Dicer promoter activity was evaluated with luciferase reporter assays. B, Schematic and primer sets of the CpG‐rich domain prediction in the Dicer promoter using EMBOSS software for the ChIP assay (top). The relative trimethylation levels of H3K27 on the Dicer promoter were analyzed by ChIP/qRT‐PCR (bottom). C, Relative binding levels of EZH2 on the Dicer promoter. D, F, The mRNA and protein expressions of EZH2 and Dicer were analyzed by qRT‐PCR (left) and Western blotting (right). E, G,, Luciferase reporter assays demonstrating Dicer promoter activity (left). Cell viability was analyzed using the MTT assay (right). H, Relative trimethylation levels of H3K27 on the Dicer promoter. Error bars indicate the mean ± SD of at least three independent experiments. *p < 0.05, **p < 0.01; Student's *t* test. I, Expression of EZH2 in liver cancer patients. ***p < 0.001; Student's *t* test. J‐L, Correlation between Dicer and EZH2 (J), EZH2 and SOX2 (K), and EZH2 and OCT4 (L) in liver cancer patients. Correlation coefficient (*r*), sample number (*n*), and *P*‐values are shown within the box plot

We found that Dicer expression was decreased in Huh7/SR cells at the protein and mRNA levels (Figure 1A and Figure S2A), and these differences may not be due to protein and mRNA stability (Figure S2B,C). To analyze the transcriptional regulation of Dicer, series Dicer promoter reporters were constructed, and a luciferase reporter assay demonstrated that the region of the *Dicer* promoter from −1327 to −605 bp was required to maintain a lower level of *Dicer* promoter activity in Huh7/SR cells (Figure 2A). CpG‐rich domains could affect histone modifiers, such as EZH2, and catalyze H3K27me3,^25^ and we found that the region of the *Dicer* promoter from −1376 to −7 bp has a CpG‐rich domain. We designed three primer sets on the *Dicer* promoter from −1327 to −605 bp for evaluation in a ChIP assay (Figure 2B, top). The ChIP assay demonstrated that Huh7/SR cells had higher H3K27me3 levels on the F1 and F3 *Dicer* promoter regions (Figure 2B, bottom). The ChIP assay also showed that EZH2 interacted more with the *Dicer* promoter F1 and F3 regions in Huh7/SR cells (Figure 2C). Knockdown of EZH2 significantly increased Dicer expression (Figure 2D), and a luciferase reporter assay also indicated that Huh7/SR/shEZH2 cells maintained a higher level of *Dicer* promoter activity (Figure 2E, left). Knockdown of EZH2 also decreased the cell viability after sorafenib treatment (Figure 2E, right). Consistently, overexpression of EZH2 in Huh7 cells decreased Dicer expression (Figure 2F). Huh7/EZH2 cells had lower levels of *Dicer* promoter activity and increased cell viability after sorafenib treatment (Figure 2G). Moreover, genetic modulation of EZH2 positively affected the binding of H3K27me3 on the *Dicer* promoter F1 and F3 regions (Figure 2H). In liver cancer patient cohorts, we found that the sorafenib responder group expressed lower EZH2 levels than the sorafenib nonresponder group (Figure 2I). We also found that EZH2 expression was inversely correlated with Dicer and positively correlated with SOX2 and OCT4 in liver cancer patients (Figure 2J‐L). According to the Oncomine database, EZH2 expression was increased in tumor tissues compared with normal tissues and was positively correlated with clinicopathologic features (tumor grades and TNM stages) and the presence of stem cell surface markers (CD133 and CD44) and inversely correlated with Dicer levels (Figure S2D‐I).

### 3.3 EZH2 is crucial for Dicer‐mediated sorafenib sensitization and cancer stemness

**FIGURE 3:**
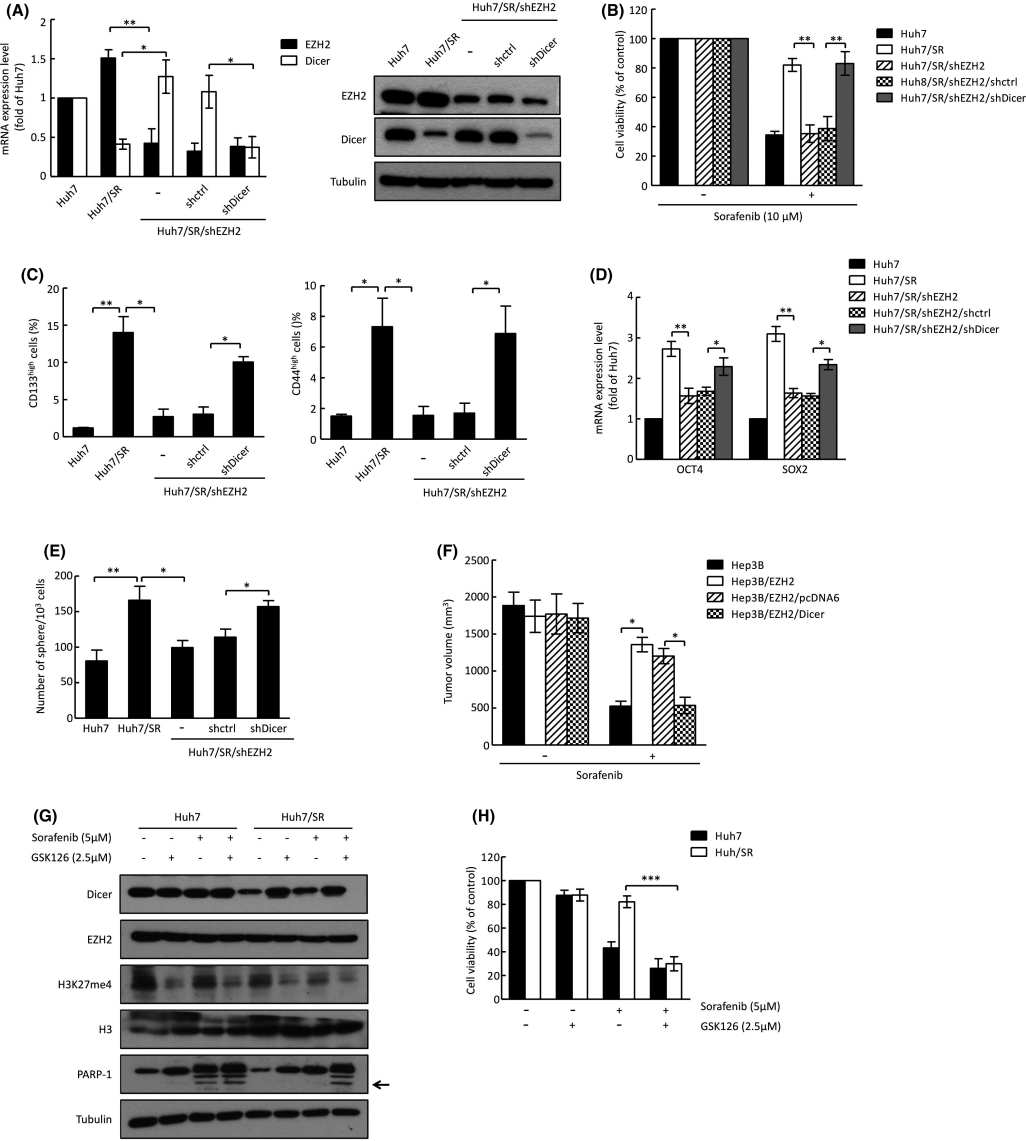
EZH2 is crucial for Dicer‐mediated sorafenib sensitization and cancer stemness. A, mRNA and protein expression of EZH2 and Dicer in the indicated cells analyzed by qRT‐PCR (left) and Western blotting (right). B, Cell viability of the indicated cells was analyzed using the MTT assay after 48 h of treatment with sorafenib. C, CD133^high^ (left) and CD44^high^ (right) populations were analyzed by flow cytometry. D, Expressions of *OCT4* and *SOX2* in the indicated cells were analyzed by qRT‐PCR. E, Sphere formation ability was examined by sphere formation assay. Error bars indicate the mean ± SD of at least three independent experiments. *p < 0.05, **p < 0.01; Student's *t* test. F, Volume of xenograft tumors formed by the indicated cells with sorafenib treatment. Error bars indicate the mean ± SD of six primary tumors. *p < 0.05; Student's *t* test. G, Protein expression was analyzed by Western blotting, and arrow indicates the cleaved form of PARP‐1. Tubulin was used as internal protein loading control. H, Cell viability was analyzed using the MTT assay. Error bars indicate the mean ± SD of three independent experiments. ***p < 0.01; Student's *t* test

To determine whether EZH2 is involved in the Dicer‐mediated regulation of sorafenib sensitivity, we transfected Dicer shRNA into EZH2‐depleted cells and found that knockdown of Dicer restored shEZH2‐supressed cell viability (Figure 3A,B and Figure S3A,B). In addition, knockdown of Dicer restored shEZH2‐suppressed cancer stemness phenotypes, including CD133^high^ and CD44^high^ populations and SOX2 and OCT4 expressions, as well as sphere formation ability (Figure 3C‐E and Figure S3C‐E). We further s.c. injected stably transfected cells (Hep3B, Hep3B/EZH2, Hep3B/EZH2/pcDNA6, and Hep3B/EZH2/Dicer) into SCID mice and determined the tumor volume after vehicle or sorafenib administration. Tumors derived from mice injected with Hep3B/EZH2 cells were more resistant to sorafenib treatment, and overexpression of Dicer abolished EZH2‐induced sorafenib resistance in our animal model (Figure 3F). Previous report has shown that the combination index (CI) of EZH2 inhibitor GSK126 and sorafenib was less than 1.0.^26^ Therefore, we cotreated Huh7 and Huh7/SR cells with GSK126 (2.5 μM) and sorafenib (5 μM) and found that combined EZH2 inhibitor GSK126 and sorafenib treatment induced cell apoptosis and decreased cell viability in Huh7/SR cells (Figure 3G,H). Collectively, these results demonstrate that both Dicer‐mediated sorafenib sensitization and cancer stemness suppression are regulated by EZH2.

### 3.4 lncRNA HOXB‐AS3 promotes the interaction between EZH2 and the Dicer promoter

**FIGURE 4:**
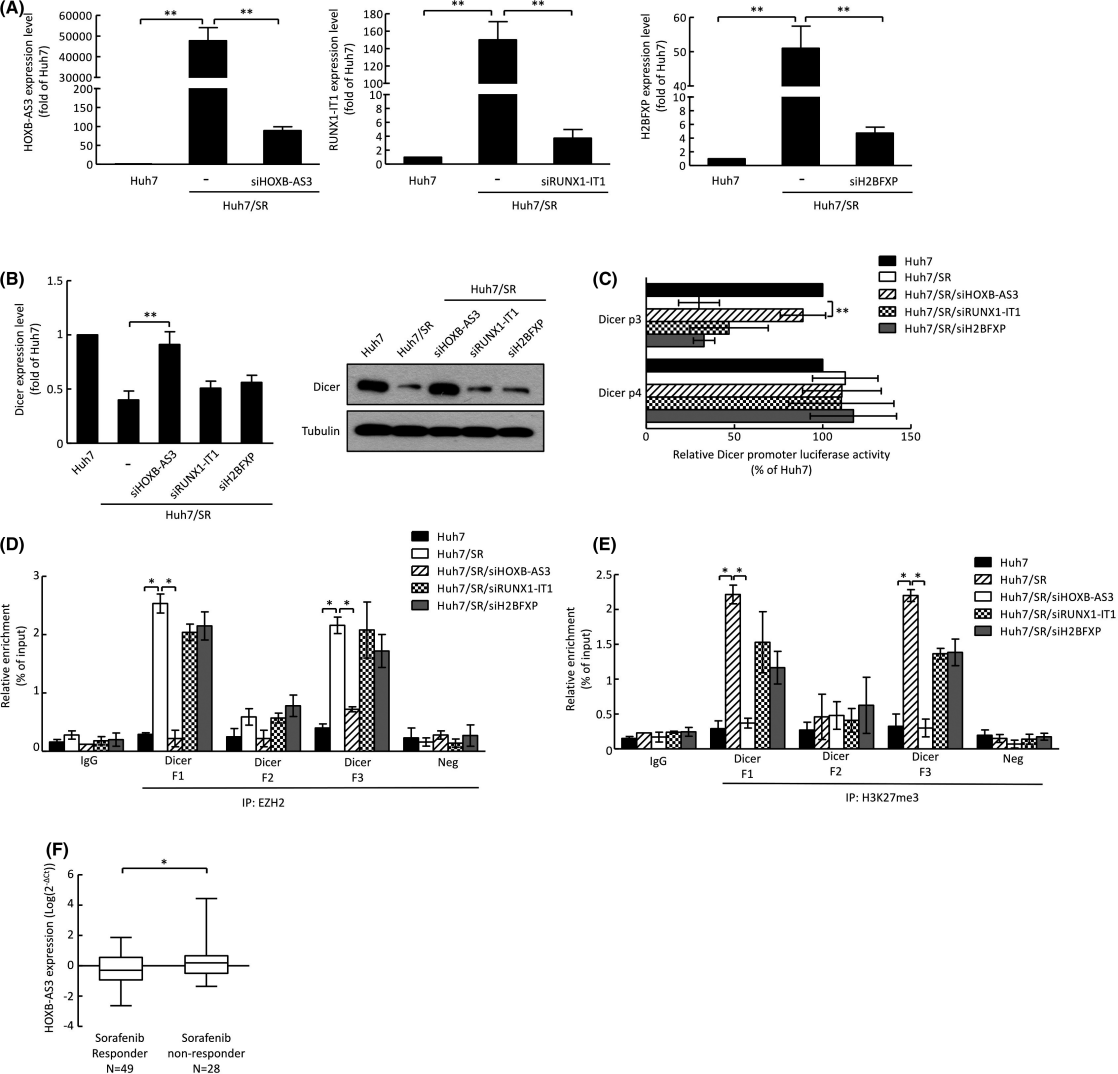
lncRNA *HOXB*‐*AS3* promotes the interaction between EZH2 and the *Dicer* promoter. A, Expression levels of lncRNAs in Huh7/SR cells with different siRNA interferences were analyzed by qRT‐PCR. B, mRNA and protein expression levels of Dicer were analyzed by qRT‐PCR (left) and Western blotting (right). C, Luciferase reporter assays demonstrate the *Dicer* promoter activity in the indicated cells. D, Relative trimethylation levels of H3K27 on the *Dicer* promoter were analyzed by ChIP/qRT‐PCR. E, Relative binding levels of EZH2 on the *Dicer* promoter were analyzed by ChIP/qRT‐PCR. Error bars indicate the mean ± SD of at least three independent experiments. *p < 0.05, **p < 0.01; Student's *t* test. F, Expression of *HOXB*‐*AS3* in liver cancer patients with different responses to sorafenib. *p < 0.05; Student's *t* test

Interestingly, several lncRNAs could serve as PcG recruiters and promote EZH2‐mediated H3K27me3.^25^ Therefore, we hypothesized that lncRNAs might contribute to EZH2‐mediated H3K27me3 on the Dicer promoter. To identify candidate lncRNAs, we performed an lncRNA microarray comparing the lncRNA expression profile between Huh7 and Huh7/SR cells, and 33 candidate lncRNAs were selected for having more than threefold differences between the cell lines. We further searched the GEO database for cancer‐related lncRNAs and identified six candidate lncRNAs (Figure S4A). RNA immunoprecipitation demonstrated that the interaction between EZH2 and three candidate lncRNAs, HOXB‐AS3, RUNX1‐IT1, and H2BFXP, was higher in Huh7/SR cells than in Huh7 cells (Figure S4B). To further validate the role of these lncRNAs, specific siRNAs were transfected into Huh7/SR (Figure 4A). Interestingly, knockdown of HOXB‐AS3, but not RUNX1‐IT1 and H2BFXP, in Huh7/SR cells increased Dicer expression at the RNA and protein levels (Figure 4B). Knockdown of HOXB‐AS3 in Huh7/SR cells maintained a higher level of Dicer promoter activity compared with control cells (Figure 4C). More importantly, we found that knockdown of HOXB‐AS3 abolished the interaction between EZH2 and two CpG‐rich regions on the Dicer promoter (Figure 4D) as well as reduced H3K27me3 on the Dicer promoter (Figure 4E). We further analyzed HOXB‐AS3 expression in patient tissues and found that sorafenib responders expressed lower HOXB‐AS3 levels than the sorafenib nonresponders (Figure 4F).

### 3.5 HOXB‐AS3–mediated Dicer suppression results in sorafenib resistance and cancer stemness

**FIGURE 5:**
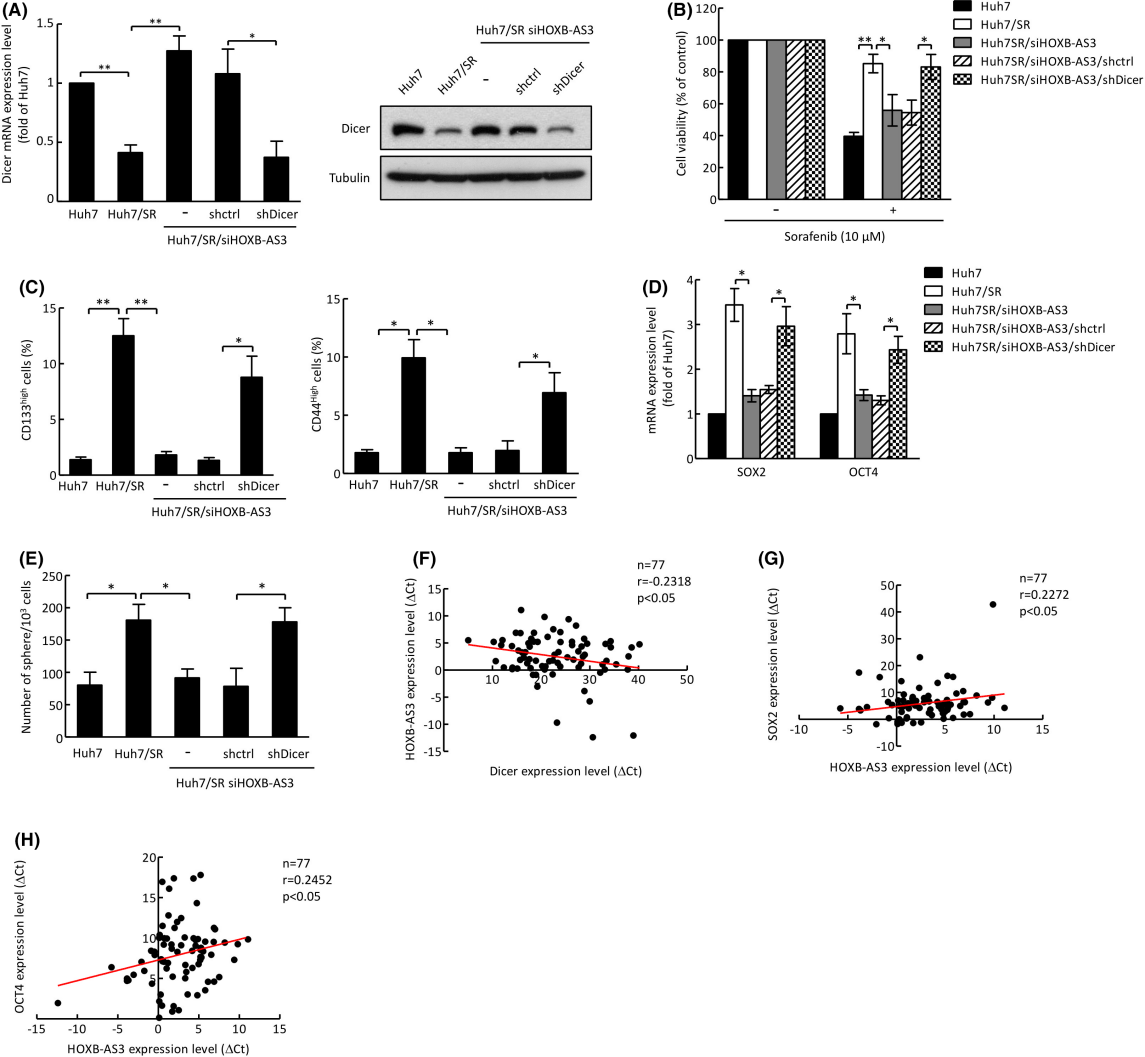
Dicer‐mediated sorafenib sensitization and cancer stemness suppression are regulated by lncRNA *HOXB*‐*AS3*. A, The expression levels of Dicer mRNA and protein were analyzed by qRT‐PCR (left) and Western blotting (right). B, Cell viability was determined using the MTT assay after 48 h of treatment with sorafenib. C, CD133^high^ (left) and CD44^high^ (right) populations were analyzed by flow cytometry. D, Expression levels of *OCT4* and *SOX2* in the indicated cells were analyzed by qRT‐PCR. E, Sphere formation ability was examined using sphere formation assay. Error bars indicate the mean ± SD of at least three independent experiments. *p < 0.05, **p < 0.01; Student's *t* test. F‐H, The correlation between *Dicer* and *HOXB*‐*AS3* (F), *HOXB*‐*AS3* and *SOX2* (G), and *HOXB*‐*AS3* and *OCT4* (H) in liver cancer patients. Correlation coefficient (*r*), sample number (*n*), and *P*‐values are shown within the box plot

To further explore whether Dicer‐mediated sorafenib sensitization is regulated by *HOXB*‐*AS3*, Dicer shRNA was transfected into Huh7/SR/si*HOXB*‐*AS3* cells (Figure 5A). We found that knockdown of Dicer in Huh7/SR/si*HOXB*‐*AS3* cells restored cell viability after sorafenib treatment (Figure 5B). In addition, *HOXB*‐*AS3* depletion reduced the CD133^high^ and CD44^high^ phenotypes, and these repressed populations were restored by shDicer (Figure 5C). Knockdown of Dicer also resulted in recovered expression of OCT4 and SOX2 as well as sphere formation ability in *HOXB*‐*AS3*–depleted cells (Figure 5D,E). To further investigate the clinical significance of *HOXB*‐*AS3*, we analyzed *HOXB*‐*AS3* expression in liver cancer patients and found that *HOXB*‐*AS3* expression was negatively correlated with Dicer expression (Figure 5F). Moreover, *HOXB*‐*AS3* expression was positively correlated with the presence of cancer stemness markers, SOX2 and OCT4, in patients (Figure 5G,H).

## 4 DISCUSSION

**FIGURE 6:**
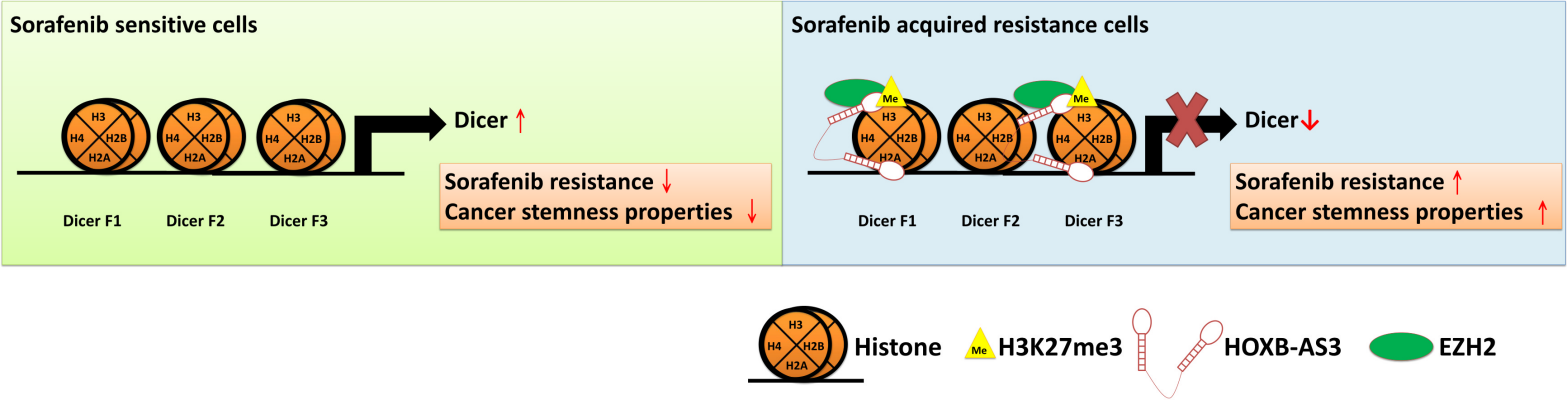
A schematic model illustrating the downregulation of Dicer via the *HOXB*‐*AS3*/EZH2 complex

Dicer is a key processing enzyme during miRNA maturation, and an increasing number of studies have indicated that miRNAs are involved in liver cancer progression and regulation of sorafenib resistance.^27^, ^28^, ^29^ Dicer expression is associated with chemoresistance in many cancer types.^30^, ^31^ For example, Dicer is a potential biomarker for predicting the clinical response to 5‐FU–based chemoradiotherapy and the overall survival in patients with oral squamous cell carcinoma.^30^ Additionally, repression of Dicer is associated with cisplatin resistance in ovarian cancer.^31^ However, the functional role and detailed regulatory mechanisms of Dicer in sorafenib sensitivity and liver cancer stemness are still unclear. In this study, we found that decreased Dicer expression resulted in sorafenib resistance and enhanced cancer stemness in liver cancer cells and patients (Table S2). The underlying mechanism by which Dicer expression has these effects was epigenetically regulated by the lncRNA *HOXB*‐*AS3*/EZH2 complex–mediated histone trimethylation (Figure 6).

Numerous miRNAs modulating cancer stemness have been identified as tumor suppressor in liver cancer. For instance, miR‐486 suppresses the expression of OCT4 and reduces cancer stemness through targeting Sirt1 in liver cancer.^32^ Downregulated miR‐137 has also been reported in sorafenib‐resistant liver cancer cells, and decreased expression of miR‐137 is associated with cancer metastasis and stemness.^33^ Similar anticancer stemness role is also observed in miR‐589. The expression of cancer stemness markers such as Oct4, Sox2, and Nanog in liver CSCs is downregulated by miR‐589.^34^ A set of miRNAs, let‐7c, miR‐200b, miR‐222, and miR‐424, have been suggested that suppress the self‐renewal of liver tumor–initiating cells and tumorigenesis.^35^ Taken together, these miRNAs identified in different studies share a similar effect on anticancer stemness. Our study also indicated that the expression of Dicer was negatively correlated with cancer stemness, and ectopic expression of Dicer could affect the properties of CSCs.

There are multiple reported mechanisms by which Dicer can be regulated: for example, Dicer is transcriptionally regulated by MITF and Tap63^12^, ^13^; the mRNA stability of Dicer is managed by miR‐103/107^14^; and the protein level of Dicer is regulated by VHL.^36^ Our study indicated that Dicer is also epigenetically regulated by H3K27me3 on the *Dicer* promoter. H3K27me3 is catalyzed by EZH2, which is a critical regulator of numerous developmental genes and controls cell differentiation and development.^37^, ^38^ Increased levels of EZH2 in cancer cells may result in tumor suppressor gene silencing, and dysregulation of EZH2 has been previously found in human liver cancer.^39^ Epigenetic silencing of Wnt antagonists by EZH2 contributes to Wnt/β‐catenin signaling activation and results in liver cancer cell proliferation.^40^ High expression of EZH2 promotes advanced non‐small cell lung cancer (NSCLC) resistance to cisplatin,^41^ and EZH2 has been suggested as a predictive marker for tamoxifen therapy in metastatic breast cancer.^42^ The aforementioned factors provide a new rationale for exploring EZH2 inhibition and increase the possibilities for a more personalized treatment approach in cancer patients. 3‐dezaneplanocin‐A (DZNep) is one of the EZH2 inhibitors and has significant antitumor activity in various cancer types, including breast, prostate, lung, liver, and brain cancer cells.^43^ SAM‐competitive inhibitors directly inhibit EZH2 by binding to its active site through competitive inhibition with methyl group donor SAM.^44^ These SAM‐competitive inhibitors also have significant antitumor activity, including growth inhibition and promotion of apoptosis.^45^, ^46^, ^47^ Our results demonstrate that EZH2 plays a critical role in these processes, and EZH2 inhibitors induce cell apoptosis and decrease cell viability in sorafenib‐resistant cells. Therefore, a combination of an EZH2 inhibitor and sorafenib is needed.

Candidate proteins include POU5F1 and yin and yang 1 (YY1),^48^ and CpG islands act as recruiters of EZH2 and are commonly associated with EZH2 target genes. However, the underlying mechanism of the interaction between EZH2 and these recruiters is unclear.^16^ Recent studies have reported that lncRNAs can be associated with the PRC2 complex and recruit EZH2 to target genes.^49^ In addition, a number of lncRNAs are dysregulated in liver cancer and affect many key signal transduction pathways involved in tumorigenesis, metastasis, prognosis, or diagnosis.^50^, ^51^, ^52^ To date, more than 200 lncRNAs encoded by four HOX loci have been found. Among these lncRNAs, HOTAIR was reported to be significantly overexpressed in HCC tissues and liver cancer cell lines.^53^ It has been reported that HOTAIR recruits the PRC2 complex to specific targets, leading to H3K27me3 and epigenetic silencing of metastasis suppressor genes.^54^ Interestingly, we found an lncRNA HOXB cluster antisense RNA 3 (*HOXB*‐*AS3*), which is also encoded by HOX loci, that is highly expressed in cells which are resistant to sorafenib and interacts with EZH2. We further demonstrated that depletion of *HOXB*‐*AS3* promoted the sensitization of liver cancer cells to sorafenib. These results provide a novel insight into the development of EZH2 inhibitors by blocking the interaction between EZH2 and lncRNAs.

In summary, we demonstrated that *HOXB*‐*AS3* epigenetically suppressed Dicer expression by recruiting EZH2 to the *Dicer* promoter and facilitated H3K27me3. We also found that repression of Dicer leads to the induction of stem‐like cell properties and enhances drug‐resistance to sorafenib in liver cancer cells. Our findings support a new regulatory mechanism of Dicer and provide a novel function for *HOXB*‐*AS3*. These findings also offer a potential strategy for targeting the *HOXB*‐*AS3*/EZH2 complex as a treatment strategy for treating liver cancer patients who have a poor response to sorafenib.

## DISCLOSURE

The authors declare that they have no competing financial interests.

## Supporting information

Appendix S1
