# Supplementary material for: Transcriptional suppression of Dicer by HOXB‐AS3/EZH2 complex dictates sorafenib resistance and cancer stemness
Source: Cancer Sci. 2022 Mar 15;113(5):1601–12. doi: 10.1111/cas.15319 (PMC9128169; doi:10.1111/cas.15319)
Supplement: Supplementary file 1 — Appendix S1 [file CAS-113-1601-s001.docx]

Table S1. Sequences and information of PCR primers for cloning and ChIP

| **Cloning primer** |
| --- |
| ***EZH2*** |
| 5’-GGGCTAGCAAAAGGAGCTGCAATT-3’ (forward) |
| 5’-GGCTCGAGAACCAAAGTAGTCACTGTT-3’ (reverse) |
| **Dicer p2277 reporter assay** |
| 5’-GGGCTAGCAAGAGGGCAACCAGA-3’ (forward) |
| 5’-GGCTCGAGGGCCTTCGAGAACCAT-3’ (reverse) |
| **Dicer p1961 reporter assay** |
| 5’-ATCTCGATTCCGACTAGCCATACTTTAAGT-3’ (forward) |
| 5’-ACTAAGCTTCTGCGTGCTGCTTCCGAGAC-3’ (reverse) |
| **Dicer p1327 reporter assay** |
| 5’-ACTAAGCTTGTTCTTTAAGCCAGCCACTTA-3’ (forward) |
| 5’-ACTAAGCTTCTGCGTGCTGCTTCCGAGAC-3’ (reverse) |
| **Dicer p605 reporter assay** |
| 5’-GGGGTGGGGGGCGGAAGTGGGTGTTTGTTA-3’ (forward) |
| 5’-ACTAAGCTTCTGCGTGCTGCTTCCGAGAC-3’ (reverse) |
| **H2BFXP siRNA** |
| 5’-GATCCCGCAGCGGCGGAGATATCTTCTTCAAGAGAGAAGATATCTCCGCCGCTGTTTTTTCCAAA-3’ (forward) |
| 5’-AGCTTTTGGAAAAAACAGCGGCGGAGATATCTTCTCTCTTGAAGAAGATATCTCCGCCGCTGCGG-3’ (reverse) |
| **HOXB-AS3 siRNA** |
| 5’-GATCCCGCTCGCACCTCTTAGGATCTTTCAAGAGAAGATCCTAAGAGGTGCGAGTTTTTTCCAAA-3’ (forward) |
| 5’-AGCTTTTGGAAAAAACTCGCACCTCTTAGGATCTTCTCTTGAAAGATCCTAAGAGGTGCGAGCGG-3’ (reverse) |
| **H2BFXP siRNA** |
| 5’-GATCCCGGAAGAGCCGAATATTTGGTTCAAGAGACCAAATATTCGGCTCTTCCTTTTTTCCAAA-3’ (forward) |
| 5’-AGCTTTTGGAAAAAAGGAAGAGCCGAATATTTGGTCTCTTGAACCAAATATTCGGCTCTTCCGG-3’ (reverse) |
|  |
|  |
| **ChIP primer** |
| **Dicer F1** |
| 5’-ATCAGCCTGCCCAATG-3’ (forward) |
| 5’-TGGCTCCTGAGCACTTAA-3’ (reverse) |
| **Dicer F2** |
| 5’-GCAGGGGCGCATAGTA-3’ (forward) |
| 5’- GGCTGGCTTAAAGAACAT -3’ (reverse) |
| **Dicer F3** |
| 5’- TCCGACCTATGCAAATGA -3’ (forward) |
| 5’- TCCCTCTGAGCACCTCC -3’ (reverse) |
| **Dicer Negative primer** |
| 5’- AGTGCCTACCCCCATACTGA-3’ (forward) |
| 5’- TTCGGTGGACTCCCTGATAA-3’ (reverse) |
|  |
| **qRT-PCR primer** |
| ***Dicer*** |
| 5’-GTCCGATGGTTCTCGAAGG-3’ (forward) |
| 5’-GCAAAGCAGGGCTTTTCA-3’ (reverse) |
| ***EZH2*** |
| 5’-CGCTTTTCTGTAGGCGATGT-3’ (forward) |
| 5’-TGGGTGTTGCATGAAAAGAATA-3’ (reverse) |
| ***FAM106A*** |
| 5’-TGTCGGCAGATTATCAAGGA-3’ (forward) |
| 5’-GGCACACCAGAATGTGTACG-3’ (reverse) |
| ***H2BFXP*** |
| 5’-AGTGCGTGACCATCACCTC-3’ (forward) |
| 5’-TGCCATATCGCACATAATGAAG-3’ (reverse) |
| ***HOXB-AS3*** |
| 5’-CCTCCCTCCAAGTCCAGTAAG-3’ (forward) |
| 5’-GGTTTCTATAGGGCCTGGAATC-3’ (reverse) |
| ***HOXB-AS3*** |
| 5’-CCTCCCTCCAAGTCCAGTAAG-3’ (forward) |
| 5’-GGTTTCTATAGGGCCTGGAATC-3’ (reverse) |
| ***LINC00221*** |
| 5’-AGGTCCTGGCTGGGAGAG-3’ (forward) |
| 5’-TGTTCTCAGAGTCTCCATTGTCTC-3’ (reverse) |
| ***LOC647859*** |
| 5’-ACACAACTGGTGGCGAGTC-3’ (forward) |
| 5’-TGTTGATCTGAAGTGATAGGTGGA-3’ (reverse) |
| ***RUNX1-IT1*** |
| 5’-AGGAAGATAGGCCCAAGCTC-3’ (forward) |
| 5’-CCTCGATTCTAAAGCCCACA-3’ (reverse) |

**Table S2. Clinicopathological features of sorafenib responders and non-responders liver cancer patients.**

| **Characteristic** | **Response to sorafenib** | | |
| --- | --- | --- | --- |
|  | **Responders(49)** | **Non-responders (28)** | ***P* value** |
| Gender (male/female) | 35/14 | 22/6 | 0.491 |
| Liver cirrhosis (Yes/No) | 18/31 | 13/15 | 0.404 |
| TNM (I,II/III,IV) | 6/33 | 8/20 | 0.709 |
| AFP (ng/ml) | 221.7 ± 544.19 | 532.1 ± 596.3 | 0.282 |
| Dicer expression (∆Ct) | 24.76 ± 3.92 | 30.81 ± 4.57 | 0.0359* |
| EZH2 expression (∆Ct) | 30.115 ± 3.72 | 26.425 ± 4.395 | 0.0745 |
| HOXB-AS3 expression (∆Ct) | 32.862 ± 5.52 | 28.792 ± 2.35 | 0.0421* |
| SOX2 expression (∆Ct) | 28.975 ± 4.88 | 29.135 ± 6.05 | 0.46 |
| OCT4 expression (∆Ct) | 28.075 ± 7.06 | 27.934 ± 4.78 | 0.795 |

**Figure S1.**

**
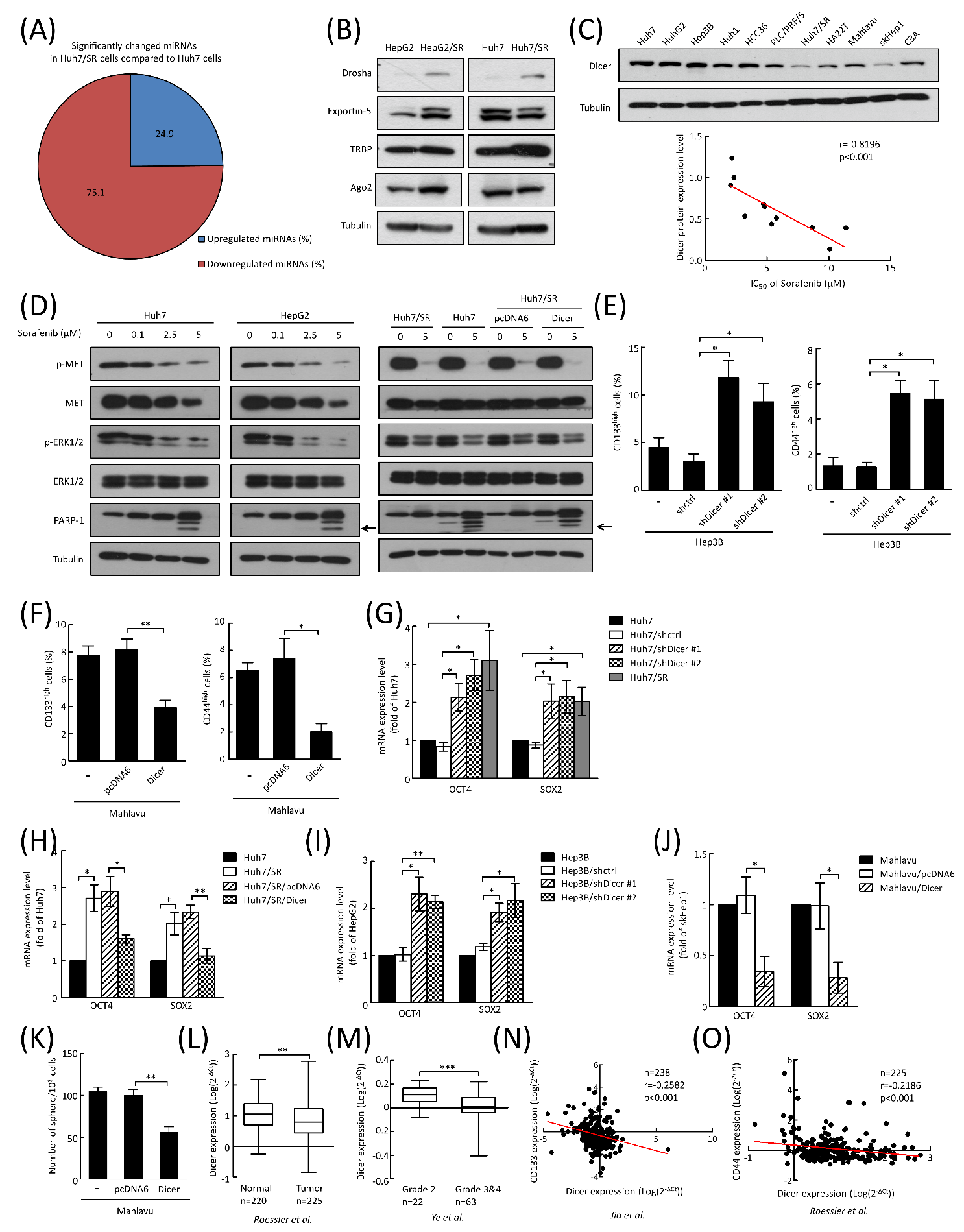
**

**Figure S1. Dicer expression negatively correlated with IC50 of sorafenib, cancer stemness and poor prognosis in liver cancer.** A, A pie chart summarizes global miRNAs differential expression in indicated cells by miRNA microarray analysis. B, The protein expression of microRNA biogenesis components (Drosha, Exportin-5, TRBP and Ago2) were analyzed by Western blotting. Tubulin was used as the internal protein loading control. C, Dicer expression was analyzed by Western blot (upper). Tubulin as the internal protein loading control. The correlation between Dicer and IC50 of sorafenib in liver cancer cell lines (lower). D, Cells were treated with sorafenib for 48hr and analyzed by Western blotting. The arrow indicated the cleaved form of PARP-1. Tubulin was used as the internal protein loading control. E, The CD133high (left) and CD44high (right) populations were analyzed by flow cytometry. Error bars shown as mean ± s.d. of three independent experiments. **p* < 0.05, ***p* < 0.01; Student’s t test. F-I, The relative OCT4 and SOX2 mRNA expression were analyzed by qRT-PCR. Error bars shown as mean ± s.d. of three independent experiments. **p* < 0.05, ***p* < 0.01; Student’s t test. J, The sphere formation ability was examined by sphere formation assay. Error bars shown as mean ± s.d. of three independent experiments. ***p* < 0.01; Student’s t test. K, Oncomine database analysis of Dicer in normal liver tissues (n = 220) and liver cancer tissues (n = 210). (Oncomine datasets: Roessler_Liver.) ****p* < 0.001; Student’s t test. L, Oncomine database analysis of Dicer in liver cancer patients with low grade (grade 2, n = 22) and high grade (grade 3&4, n = 63). (Oncomine datasets: Yer_Liver.) ****p* < 0.001; Student’s t test. M, N, The correlation between CD133 and Dicer (M), CD44 and Dicer (N) in liver cancer patients. (Oncomine datasets: Jia_Liver and Roessler_Liver.) Statistics from individual studies were obtained from the Oncomine database. The correlation coefficient (r), sample number (n) and p-values are shown within box plot.

**Figure S2.**


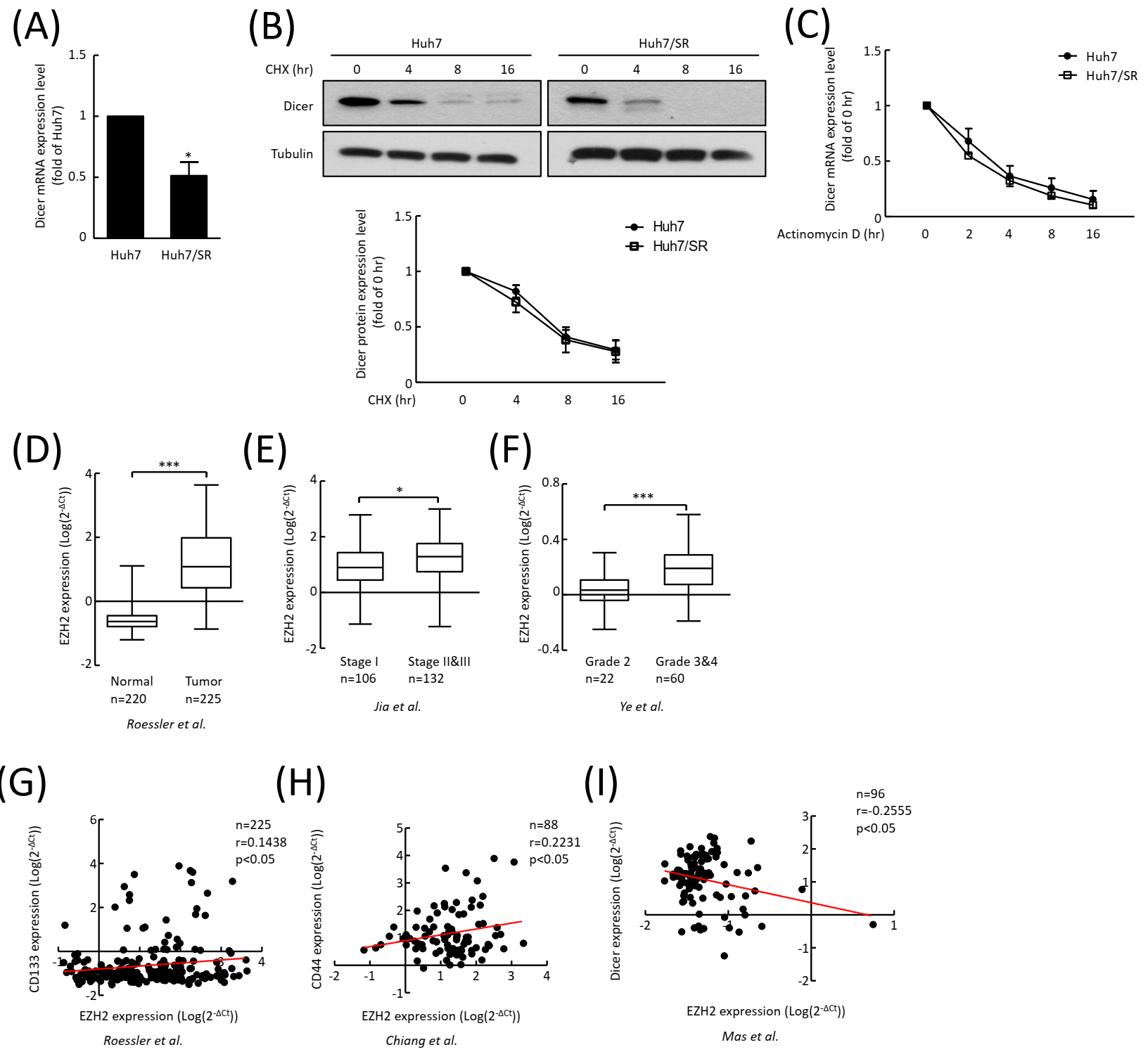


**Figure S2. The mRNA and protein stability of Dicer in Huh7 and Huh7/SR cells and the correlation between clinicopathological features and EZH2 expression in liver cancer patients.** A, Dicer mRNA expression was analyzed by qRT-PCR. The qRT-PCR data were normalized to the level of GAPDH. Error bars shown as mean ± s.d. of three independent experiments. **p* < 0.05; Student’s t test. B, The protein level of Dicer was examined by Western blot in Huh7 and Huh7/SR cells treated with cycloheximide (50 μg/ml, CHX) at indicated time points. Tubulin as the internal protein loading control (upper). The protein level of Dicer was quantified by ImageJ software (lower). C, The mRNA level of Dicer was analyzed by qRT-PCR in Huh7 and Huh7/SR cells in the presence of actinomycin D (1 µg/ml) at the indicated time points. The qRT-PCR data were normalized to the level of GAPDH. D, Oncomine database analysis of the levels of EZH2 in normal liver tissues (n = 220) and liver cancer tissues (n = 210). (Oncomine datasets: Roessler_Liver.) ****p* < 0.001; Student’s t test. E, EZH2 expression in liver cancer patients with stage I (n = 106) and stage II & III (n = 132). (Oncomine datasets: Jia_Liver.) **p* < 0.05; Student’s t test. F, EZH2 expression in liver cancer patients with low grade (grade 2, n = 22) and High grade (grade 3&4, n = 63). (Oncomine datasets: Ye_Liver.) ****p* < 0.001; Student’s t test. G-I, The correlation between EZH2 and CD133 expression (G); EZH2 and CD44 expression (H); Dicer and EZH2 expression (I) in liver cancer patients. (Oncomine datasets: Roessler_liver, Chiang_Liver and Mas_Liver.) Statistics from individual studies were obtained from the Oncomine cancer database. The correlation coefficient (r), sample number (n) and p-values are shown within each box plot.

**Figure S3.**


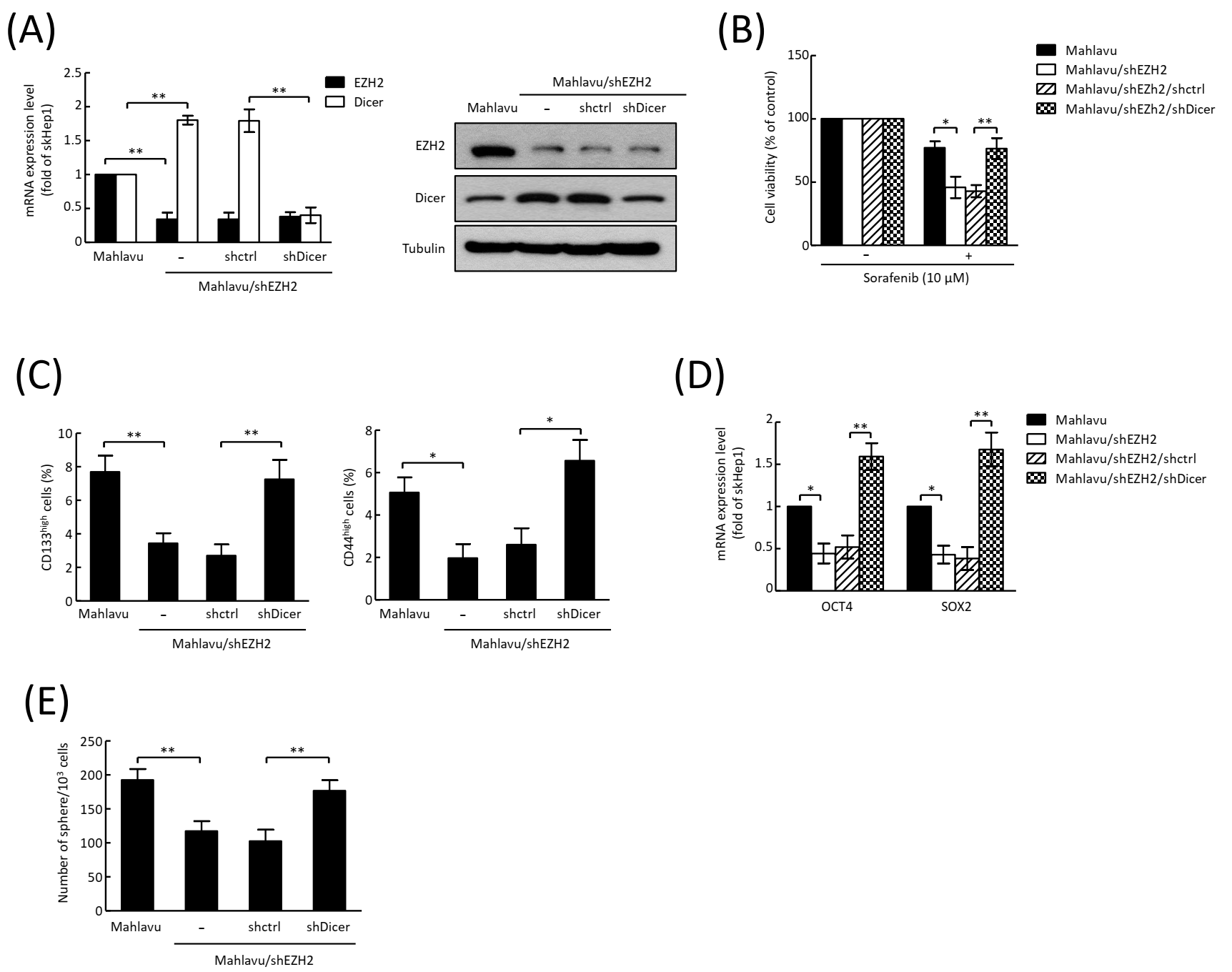


**Figure S3. Dicer-mediated sorafenib sensitization and cancer stemness suppression are regulated by EZH2.** A, The mRNA and protein expressions of EZH2 and Dicer were analyzed by qRT-PCR and Western blot. The qRT-PCR data were normalized to the level of GAPDH. Tubulin as the internal protein loading control. Error bars are shown as mean ± s.d. of three independent experiments. ***p* < 0.01; Student’s t test. B, Cell viability was analysed by MTT assay after treatment with sorafenib for 48 hours. Error bars are shown as mean ± s.d. of three independent experiments. **p* < 0.05, ***p* < 0.01; Student’s t test. C, The CD133high (left) population and CD44high (right) populations were analyzed by flow cytometry. Error bars shown as mean ± s.d. of three independent experiments. **p* < 0.05, ***p* < 0.01; Student’s t test. D, The relative OCT4 and SOX2 mRNA expression was analyzed by qRT-PCR. The qRT-PCR data were normalized to the level of GAPDH. Error bars are shown as mean ± s.d. of three independent experiments. **p* < 0.05, ***p* < 0.01;Student’s t test. E, The sphere formation ability was examined by sphere formation assay. Error bars are shown as mean ± s.d. of three independent experiments. **p < 0.01; Student’s t test.

**Figure S4.**


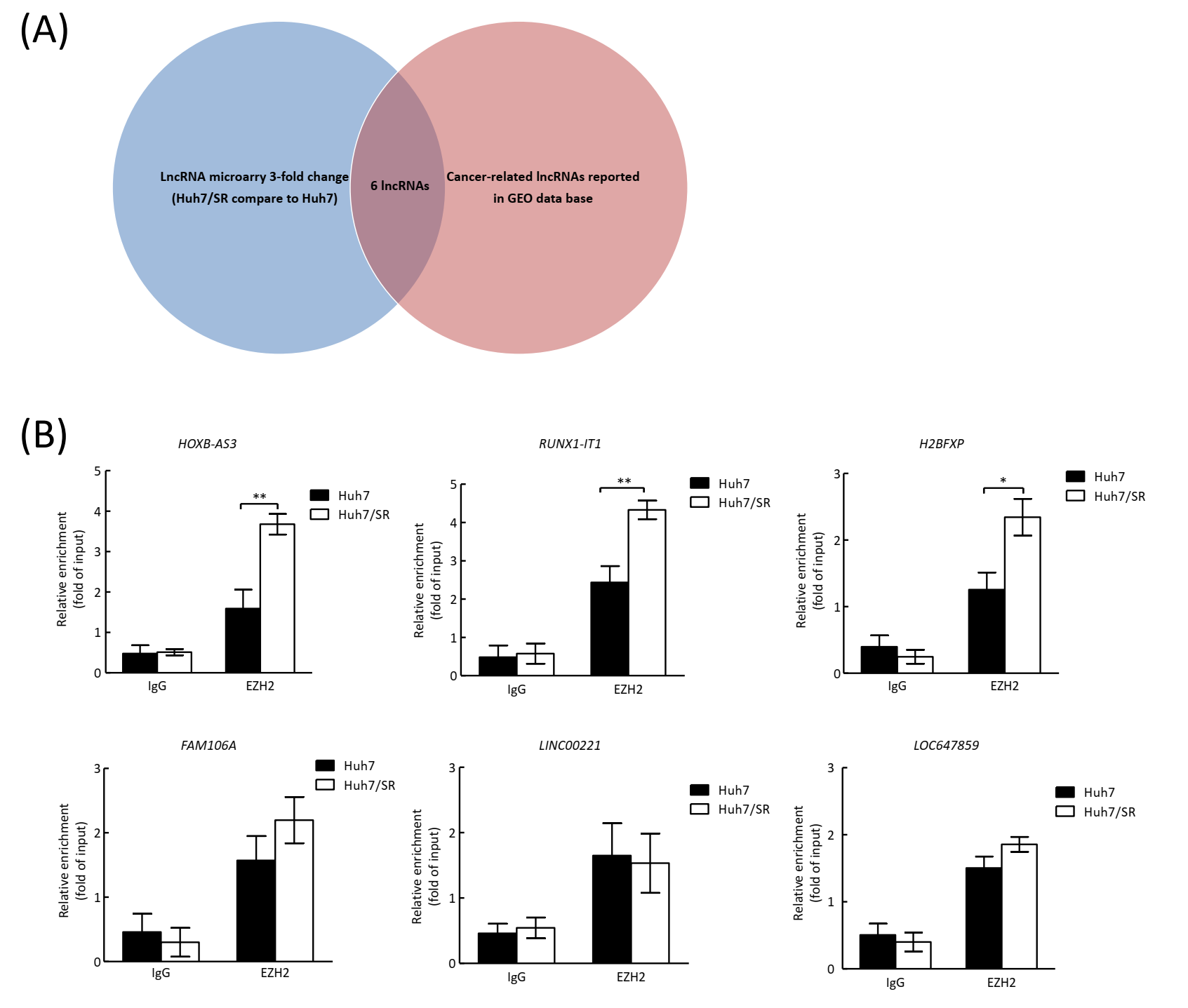


**Figure S4. Candidate lncRNAs were identified by lncRNA microarray.** A, A Venn diagram showed that 6 lncRNAs were related to cancer in GEO data base and differential expression between Huh7 and Huh7/SR cells by lncRNA microarray analysis. B, RNA Immunoprecipitation (RIP) was performed using antibodies against IgG and EZH2. The enrichment of HOXB-AS3, RUNX1-IT, H2BFXP, FAM106A, LINC00221 and LOC647859 was measured by qRT-PCR. Error bars are shown as mean ± s.d. of three independent experiments. **p* < 0.05, ***p* < 0.01; Student’s t test.
